# Perspectives of Clinical Teaching Fellows on preparedness for practice: a mixed-methods exploration of what needs to change

**DOI:** 10.1080/10872981.2021.1976443

**Published:** 2021-09-17

**Authors:** Rory Morrice, Olivia Buckeldee, Kathleen Leedham-Green

**Affiliations:** Faculty of Medicine, Imperial College London, London, United Kingdom

**Keywords:** Clinical Teaching Fellow, medical education - undergraduate, preparedness, medical curriculum, transition

## Abstract

Supporting medical students in their transition to newly qualified doctor is an important educational priority. Clinical Teaching Fellows (CTFs), as both recent graduates and trained educators, are uniquely positioned to suggest curricular enhancements to support preparedness for practice. Our mixed-methods approach involved CTFs across eight UK teaching hospitals. We conducted five activity-oriented focus groups to explore what CTFs felt needed to change to increase preparedness for practice. We analysed these focus groups to create a dataset of their suggestions followed by a survey. The survey invited CTFs to rate and rank these suggestions in relation to their own self-rated preparedness for practice, with qualitative insights into their choices. We explored commonalities and differences between high and low confidence participants, with findings qualitatively illuminated. 24 CTFs attended focus groups from which we identified 28 curriculum items and 10 curriculum agendas. We collected 23 complete survey responses. All confidence groups rated communicating with colleagues and managing working life as unmet needs, whereas core clinical competencies such as history and examination were well met. Participants with low confidence identified more complex clinical competencies including clinical decision making, task prioritisation and end-of-life care as unmet needs, with decision making and prioritisation being the most important. Confident graduates rated higher professional competencies such as quality improvement, career planning and education as unmet needs but of low importance. Graded transition of responsibility was the highest ranked curriculum agenda. Qualitative insights included suggestions for how learning in clinical environments could be enhanced. Our findings suggest that transitioning from student to newly qualified doctor could be supported by graded entrustment and enhanced shadowing opportunities. Other recommendations include prioritising more complex clinical competencies, identifying wellbeing as part of preparedness for practice, equipping students to communicate with colleagues and aligning higher professional competencies with learners’ needs.

## Background

Doctors face multiple transitions throughout their training [1]. These periods of change and discontinuity require trainees to adapt to new environments and expectations [[2]. The transition from student to doctor can provide opportunities for transformative learning and professional identity formation, however, if poorly managed, may serve as a barrier to personal growth and development [[3]. Supporting medical students in their transition to life as a doctor is a challenge faced internationally by medical schools.

In the UK, trainees begin their first year of clinical practice as an intern doctor, named Foundation Year 1 (FY1), within the National Health Service (NHS). They may be working in new areas of the country and in unfamiliar hospitals. In order to successfully navigate this discontinuity, the UK’s regulator for the medical profession, the General Medical Council (GMC), outlines preparedness for practice as an educational priority for medical schools [4]. Targeted strategies to prepare graduates for the transition to clinical practice, such as shadowing periods, have been developed. While these innovations can improve students’ self-reported preparedness, they do not seem to alter the prominence of stress and anxiety associated with this transition [5]. Self-reported unpreparedness amongst newly qualified doctors remains a significant issue with many graduates still perceiving this as a barrier to effective clinical practice and subsequent implications on the quality of patient care [5–8,9].

In the past decade, Clinical Teaching Fellow (CTF) posts have become popular in the UK [10]. CTFs are postgraduate doctors that have completed at least two years of clinical practice. They are employed, typically for one year, to teach in the clinical workplace. The GMC requires that they are trained and supported in educational practice. CTFs can provide fresh perspectives on established educational practices [11] and many develop innovative strategies to support preparedness for practice [12–14].

Medical students rate CTF-led education highly, which may relate to their near-peer status and their ability to understand students’ learning needs [van Heerden et al., 15]. This fresh perspective may also help to address the previously documented discordance between the views of learners and members of higher education faculty on what is needed to enhance preparedness for practice (Colbert-Getz et al., 2016). Given CTFs unique position as trained educators with lived experience of their own recent transition from student to doctor, we wanted to explore their perspectives on undergraduate medical education with a focus on improving the transition to clinical practice.

### Aim

To explore CTF’s perceptions of their own unmet needs at the transition from student to doctor, and their perspective on what needs to change in undergraduate medical education to enhance preparedness for practice.

## Methods

### Participants and setting

We invited all 52 CTFs employed within the last two years across eight NW London teaching hospitals to participate, 24 of which were currently actively employed in CTF posts. Participants either had a prior educational qualification or had attended training accredited at the level 2 professional standards of the Academy of Medical Educators **[**16**]**.

### Methodology

Our assumptions are that although there may be real differences in how prepared graduates are for practice, we can only approach an understanding of this through the interpretation of subjective experience.

**Figure 1.**
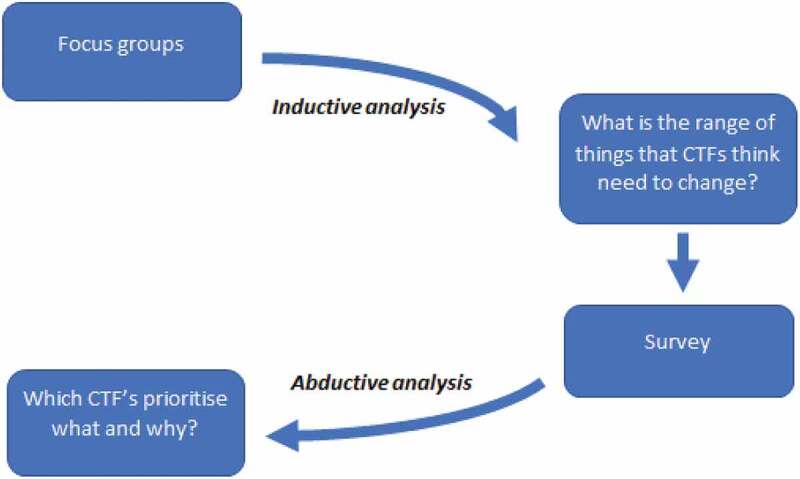
Summary of research

We employed a mixed methods research process and an inductive approach to analysis. We began with focus groups to identify the range of factors that CTFs felt needed to change, followed by a survey that invited participants to sort and rank those factors according to their own experience and to provide additional qualitative insights into their reasoning. The synthesis of qualitative and quantitative data was achieved using the focus group data to create the survey and using the additional qualitative data from the survey to illuminate our quantitative results. This multi-stage process is illustrated in (Figure 1).

### Research ethics

Ethical approval was provided by Imperial College London Education Ethics Review Process EERP1819-006. All data were collected anonymously and any potentially identifying information redacted prior to analysis.

### Research team

The research team comprised two current CTFs (Researchers OB and RM) and an educational research fellow (KLG).

## Stage 1: activity-oriented focus groups

KLG ran five activity-oriented focus groups [17] at a CTF research workshop. Participants were asked the following question: ‘Based on your own experience, what would you change about your undergraduate curriculum to increase your preparedness for practice?’ Participants were invited to write their individual responses on sticky notes and then to compare and discuss their responses in groups with 4–5 peers. Each of the five groups then organised their responses into themes on a flip-chart sheet, adding or combining items as they progressed. Researchers RM and OB collated the responses and identified discrete items which were grouped into themes. The themes and items were collaboratively refined and checked back against the underlying focus group data. This collaborative review process sorted the discrete items into ‘curriculum items’, relating to topics that participants felt were not adequately covered within their own undergraduate education, and ‘curriculum agendas’, relating to educational processes that they felt needed to change.

## Stage 2: survey

### Survey design

We converted curriculum items and agendas into a Qualtrics survey (Appendix 1). The survey invited participants to retrospectively rate their own ‘preparedness for practice’ at graduation and then after their induction, using a 10-point scale. It also asked participants to estimate the number of months it took in their first year of clinical practice to feel ‘prepared’.

For each curriculum item participants were asked ‘Would more teaching on these/knowing more about these have helped you feel better prepared for practice as an FY1?’ and invited to sort each item into one of four groups: needs were met, not really needed, unmet need, or unsure. For each curriculum agenda participants were asked ‘As a teaching fellow, what do you think would help prepare students for practice as an FY1?’ and invited to sort statements into four groups: agree, this is already about right, wouldn’t help and unsure. Participants were invited to provide qualitative insights as they sorted curriculum items and agendas in each theme. Finally, to identify participants’ perspectives on the relative importance of each item, we invited them to rank their unmet needs and agreed curriculum agendas according to their own experience and viewpoint. Demographic data were collected relating to participants’ graduation year, medical school, entry type and course design.

We invited two CTFs to review the survey for clarity, usability, acceptability, and inclusivity which resulted in a reduction in the number of free text boxes, changes to the question wording, removal of similar items and modifications to the demographic questions.

### Survey distribution

We distributed the survey to participants by email and monthly newsletter between November 2019 to February 2020.

### Data analysis

We combined the 3 measures of self-reported preparedness for practice to create 4 roughly equal groups of participants: very confident, confident, medium confidence, low confidence (Table 1). We counted how often each item was sorted as an unmet need or agreed curriculum agenda (frequency) and calculated the median rank (MR), and interquartile range (IQR) of ranking for each item in total and across confidence groups. We used the median number of curriculum items and curriculum agendas ranked by participants to create a weighted score to reflect the median rank (MR) for each item, with unmet needs scored from 1 (highest ranked) to 10 (lowest ranked) and curriculum agendas scored from 1 to 6. The raw data and analysis were reviewed collaboratively by all authors and key patterns and differences discussed. Qualitative survey data were collated within each of the survey themes and selected for inclusion in our results table based on their ability to triangulate or illuminate our quantitative results.

**Table 1.** Respondent characteristics and confidence categories

Response ID	Year of Graduation	Medical School*	Entry Type**	Course Type***	Time to Confidence	Confidence category
1	2011	Russell Group	Standard	Traditional	1–2 months	Very Confident (VC) Median unmet needs (range) 5.0 (2–10) Median curriculum agendas (range) 6.5 (2–8) Average confidence on graduation 8.7/10 Average confidence after induction 8.8/10
2	2017	Non-Russell Group	Standard	Integrated	1–2 months	
3	2017	Non-Russell Group	Extended	Integrated	1–2 months	
4	2017	Non-Russell Group	Graduate	PBL	1–2 months	
5	2011	Non-Russell Group	Standard	PBL	1–2 months	
6	2011	Russell Group	Standard	Integrated	1–2 months	
7	2016	Russell Group	Standard	Integrated	3–5 months	Confident (C) Median unmet needs (range) 10.0 (4–17) Median curriculum agendas (range) 5.5 (4–7) Average confidence on graduation 7.3/10 Average confidence after induction 7.5/10
8	2017	Russell Group	Standard	Integrated	3–5 months	
9	2016	Russell Group	Standard	Traditional	3–5 months	
10	2016	Russell Group	Standard	Traditional	3–5 months	
11	2017	Russell Group	Standard	Traditional	3–5 months	
12	2015	Overseas	Standard	Integrated	3–5 months	
13	2016	Russell Group	Extended	Integrated	3–5 months	Medium confidence (MC) Median unmet needs (range) 9.5 (5–13) Median curriculum agendas (range) 6.5 (4–8) Average confidence on graduation 4.8/10 Average confidence after induction 6.8/10
14	2016	Russell Group	Standard	Traditional	3–5 months	
15	2017	Overseas	Graduate	Traditional	3–5 months	
16	2014	Russell Group	Standard	Traditional	3–5 months	
17	2015	Non-Russell Group	Standard	Traditional	3–5 months	
18	2016	Non-Russell Group	Standard	Integrated	≥6 months	
19	2008	Non-Russell Group	Standard	Traditional	≥6 months	Low confidence (LC) Median unmet needs (range) 13.0 (12–16) Median curriculum agendas (range) 6 (4–8) Average confidence on graduation 4.6/10 Average confidence after induction 3.0/10
20	2017	Russell Group	Standard	Traditional	≥6 months	
21	2015	Russell Group	Standard	Traditional	≥6 months	
22	2010	Russell Group	Graduate	Traditional	≥6 months	
23	2017	Russell Group	Standard	Traditional	≥6 months	

* **Medical school**: Russell Group = medical schools are generally linked to longer established universities and are considered higher status schools; Non-Russell Group = medical schools are typically linked to newer universities; Overseas = studied medicine outside the UK

** **Entry type**: Extended = longer course typically involving an additional pre-medicine year; Graduate = shortened course for students with a previous university degree or professional experience; Standard = standard length course

*** **Course type**: PBL = course based on problem-based learning and other active pedagogies; Traditional = typically two years of biomedical sciences lectures followed by three years of clinical training; Integrated = biomedical sciences and clinical training across all years.

## Results

### Stage 1: focus group results

24 of the 52 CTFs attended the activity-oriented focus groups, with representation from all eight networked teaching hospitals. We identified 38 discrete items following collaborative review of focus group responses. The 38 discrete items were further categorised into 28 ‘curriculum items’, relating to topics that participants felt were not adequately covered within their own undergraduate education, and 10 ‘curriculum agendas’, relating to educational processes that they felt needed to change. These are listed in (Tables 2 and Tables 3).

**Table 2.** Unmet curriculum items

Theme	Curriculum Items	How many CTFs rated as unmet need (n = 23)	Median rank (1–10, 1 = most important)	Interquartile Range	Example quotes
NHS Structure	NHS structure and funding (NS1)	11	9	4	‘All of these things are good to learn as you plan and progress through your career, but I don’t think any are actually needed to be a decent FY1’ [Respondent 1 – VC] ‘We had very little in the way of career planning at undergraduate level but I’m not sure it would have helped me feel better prepared for practice at FY1’ [Respondent 6 – VC]
	Career planning (NS2)	11	8	2	
	Postgraduate portfolios (NS3)	11	8	3	
	Understanding roles of allied HPs (NS4)	6	8	2.25	
	Staff hierarchy (NS5)	2	6	-	
Managing Working Life	Work life balance (WL1)	12	6	4.5	‘Well-being was something that was never ever spoken about. It seems crazy that we were dropped in to intensive on call rotas with little support at times and no one had ever said “this could impact on your mental well-being”’ [Respondent 10 – C] ‘Mental health and coping with working shift patterns/work-life balance were not discussed when I was at medical school – Q&A sessions would have been invaluable prior to starting FY1’ [Respondent 23 – LC]
	Wellbeing (WL2)	14	5.5	7.5	
	Out of hours working (WL3)	16	7	6.5	
	Q&A sessions with doctors (WL4)	6	8	7.75	
Communication Skills	Patients/relatives (CS1)	3	3	-	‘I was lucky that my undergraduate course had a lot of role plays in the final year on difficult relatives, patients, colleagues … It was only after graduating that I realised how important all this was … Valuable learning but I probably didn’t appreciate it at the time as I was so exam focused’ [Respondent 21 – LC] ‘I have highlighted the difficult colleagues as most important as I felt this had the greatest impact on my work during FY1 and still find it difficult to this day’ [Respondent 8 – C]
	End of Life care (CS2)	7	5	3	
	Breaking bad news (CS3)	2	4.5	-	
	Difficult colleagues (CS4)	11	4	5.5	
Job Practicalities	Referrals (JP1)	15	3	1	‘Learning trust processes added efficiency to knowledge; without efficiency, it felt like competency was continually questioned by simple tasks like prescribing or discharge paperwork’ [Respondent 3 – VC] ‘I felt most unprepared for the hands-on stuff, despite having lots of “hands on time” at university. It was the stuff I always saw but never actually got to do – making referrals, answering bleeps, working on-call. Being involved in this with a degree of responsibility would have been a big help. FY1 is really difficult at times.’ [Respondent 10 – C]
	IT systems (JP2)	12	4	5.5	
	Task prioritisation (JP3)	6	2	1	
	Documentation (JP4)	5	3	3	
	Prescribing (JP5)	3	6	-	
	Bleep training (JP6)	9	4	3	
Clinical Competencies	Handover (CC1)	6	6.5	4.75	‘I wish I had been pushed into the role of junior doctor in my final year a bit more (I was generally just opening and closing curtains on ward rounds). I needed to be making decisions on diagnosis and management whilst I was still fully supervised so that I could get feedback.’ [Respondent 22 – LC] ‘We had plenty of history taking and handover training which was helpful. We also had the knowledge to recognise the “acute presentations” when patients were really unwell. These were the easy ones, you knew you needed senior help and you got it pretty fast. The training that was missing was what to do when you aren’t sure whether the patient is “really sick” or not’ [Respondent 12 – C]
	Assessing acutely unwell patients (CC2)	1	5	-	
	Clinical reasoning and management plans (CC3)	7	1	4.5	
	History taking (CC4)	0	-	-	
	Presenting at an MDT (CC5)	11	8	3	
Professional Competencies	Serious Incident reporting (PC1)	9	7	5	‘I am not sure that teaching on serious incident reporting and QI skills would have necessarily helped in day to day practice; I think this is something that can be learned on the job and from senior colleagues.’ [Respondent 23 – LC] ‘Quality improvement was not covered and suddenly we are expected to be able to lead and perform a project in a busy FY1 year when we are trying to cope with everything else’ [Respondent 10 – C]
	Quality Improvement Skills (PC2)	9	6	7	
	Teaching (PC3)	7	6	4.5	
	Teamwork (PC4)	4	4	3.25

**Table 3.** Curriculum agendas

Curriculum Agenda	Frequency (n = 23)	Median rank (1–6, 1 = most important)	Interquartile Range	Example quotes
More biomedical learning (BM)	2	4.5	-	‘I actually think all the multiple-choice questions on weird bits of biomedical knowledge stopped me getting to grips with learning to be a good doctor. You can’t learn those answers from being on the wards – you have to go to the library.’ [Respondent 22]
More knowledge MCQs (MCQ)	3	5	-	‘Having done short and long answer questions in my first year and my intercalated degree, they prompt and encourage a much greater depth of learning and an appreciation of uncertainty in clinical practice which I think has been lost, largely due to there now being clearly identified right and wrong answers [in MCQs]’ [Respondent 6]
Assessments aligned to foundation competencies (AF)	19	5	2.5	‘I was never assessed in a CEX or CBD format which would have been useful seeing as they’re the crux of foundation assessment’ [Respondent 10]
Transition of responsibility with graded responsibilities (TR)	21	2	2.25	‘I was lucky to have a transition period, but did not have any responsibility. The shift from zero to full responsibility as you start work is scary.’ [Respondent 10] ‘I think transition of responsibility would be difficult logistically but also emotionally (to be ready) unless you had more intense clinical placements throughout medical school.’ [Respondent 8]
More time working in FY1 shift patterns (SP)	18	3	2	‘I shadowed FY1s/most junior member of the team during my final year of medical school and the experience was invaluable … a good FY1 isn’t the smartest, it is the one who can get things done and moving’ [Respondent 5]
Junior doctor mentors (JM)	19	5	2.75	‘expecting a clinician to balance both their work load and added teaching shouldn’t be a given without discussion with that individual’ [Respondent 17]
Longer transition period following finals (TP)	16	3	2	‘I really valued my TTFY1 placement – it was post finals so I could just concentrate on preparing for work as a doctor. We had a portfolio that was assessed on our work during TTFY1 – including discharge summaries, patients clerked, referrals etc. – I think assessment in this way ensured students were still motivated to attend TTFY1’ [Respondent 8]
Earlier exposure to clinical environments (CE)	11	3	3	‘You need a really good grounding before you can be useful/involved on the wards. If you are on placement and you can’t engage because you don’t know anything you just feel a bit like a lemon’ [Respondent 22]
Autonomy in curriculum design (A)	11	6	0.75	‘Autonomy is nice with student selected components, but I feel if I had to choose too much, I would feel like I’m missing big areas of core knowledge’ [Respondent 10]
Simulation based learning (S)	20	3	3.25	‘Simulation would allow us to explore many of the gaps (such as conflict with seniors, patients or uncertainty) in a realistic but controlled environment.’ [Respondent 12] ‘I found [simulation] a bit baffling and it didn’t really reflect the complexities of the clinical working environment. I would have liked simulated scenarios in the actual workplace’ [Respondent 21]

‘Curriculum items’ were further grouped into six sub-themes relating to:

- Knowing how the NHS works (careers, roles, structures etc.)
- Managing working life (wellbeing, peer support etc.)
- Communicational and relational (difficult colleagues, breaking bad news, communicating with relatives etc.)
- Job practicalities (bleeps, documentation, referrals, IT systems etc.)
- Clinical competencies (clinical decision making, history taking, handover, acute clinical scenarios etc)
- Professional competencies (teaching, quality improvement, incident reporting, team working etc.)

‘Curriculum agendas’ related to:

- Balancing biomedical learning with the practical dimensions of clinical learning (2 items)
- Ensuring more learning is situated in the clinical workplace
- Aligning undergraduate teaching and assessment to foundation competencies
- Graded transition of responsibilities
- Near-peer mentoring
- Longer shadowing opportunities
- Out-of-hours shadowing opportunities
- Student involvement in curriculum design
- More simulation-based learning

### Stage 2: survey results

#### Respondent characteristics

We received 30 responses, 23 of which were complete. The respondent characteristics are shown in (Table 1). There were no significant differences in confidence groups between participants graduating from a higher status UK university (Russell Group) or not. Further, there were no difference in confidence according to whether the participant was a graduate-, standard- or extended-entry student (described in Table 1). However, a χ^2^ test suggests that significantly more participants in the two higher confidence categories graduated from integrated or problem-based learning (PBL) courses, with more from traditional courses in the medium/low confidence groups (p = 0.01). However, this requires careful interpretation given the small sample size.

#### Curriculum items

**Figure 2.**
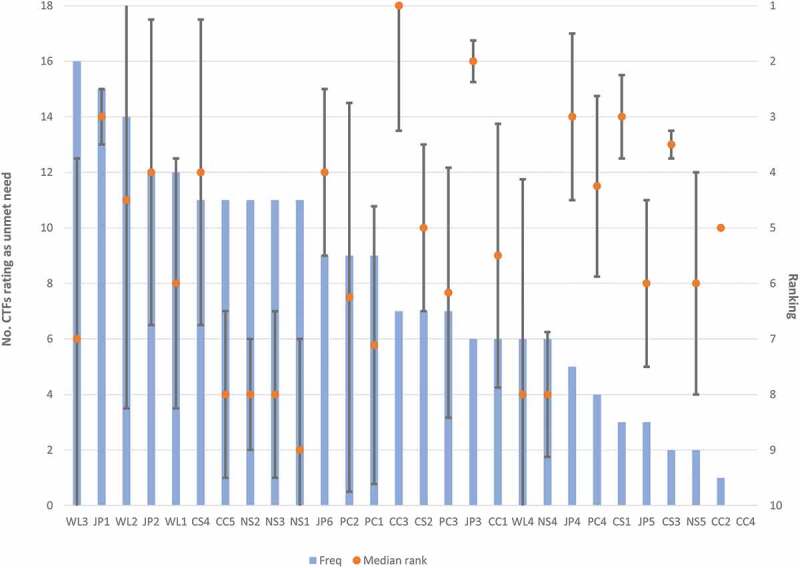
Unmet curriculum items

The number of times each curriculum item was ranked as an unmet need (frequency), its perceived importance (median rank), and inter-quartile range are shown in (Figure 2). (Table 2) presents this data with illustrative qualitative insights alongside each theme. The number of participants in each confidence group is small, therefore we haven’t made assertions about significance or effect size. However, salient observations can be made and absolute numbers and median ranking are reported where appropriate.

#### Comparing curriculum items between confidence groups

Higher confidence groups sorted fewer unmet curriculum items than lower confidence groups (VC vs. LC group median frequency 5 vs. 13). There was a wider range in the very confident group but a consistently large number of unmet needs in the low confidence group (VC 2–10 vs. LC 12–16).

##### Low confidence groups: Complex clinical competencies are highly ranked unmet needs

4/5 participants who ranked ‘Clinical Reasoning’ as their most important unmet need (MR 1) were in either the low or medium confidence groups. Lower confidence participants felt that not having the opportunity to practise formulating management plans was an important barrier to their preparedness.

I wish I had been pushed into the role of junior doctor in my final year a bit more (I was generally just opening and closing curtains on ward rounds). I needed to be making decisions on diagnosis and management whilst I was still fully supervised so that I could get feedback. [Respondent 22 - LC]

‘End of Life Care’ was ranked as an unmet need by 4/5 in low confidence group but only 1 participant in any other confidence group. ‘Task Prioritisation’ was a frequently and highly ranked unmet need in the low confidence group (n = 3/5, MR 2) but was not considered unmet by any participants in the high confidence group.

##### High confidence groups: Higher order professional competencies considered unmet

Participants in the very confident or confident groups frequently rated items within the Professional Competencies theme as an unmet need (n = 9/12). However, no-one in the low confidence group rated ‘Quality improvement skills’, ‘Teaching’, ‘Teamwork’ as an unmet need. Although higher professional competencies were frequently rated as unmet needs by higher confidence participants, they considered these items to be of low importance for preparedness for clinical practice.

There are more important priorities in my mind at this stage [Respondent 8 - C]

… the top-ranked options for me are things that you need to be able to do on day 1 FY1 and very few people I’ve met felt confident doing so. [Respondent 9 - C]

Despite this, these items appeared to be a perceived deficit in the training of higher confidence participants, whereas lower confidence groups did not appear concerned by these.

Poor serious incident training at induction meant that I still don’t know how to properly report serious incidents! [Respondent 4 – VC]

Quality improvement was not covered and suddenly we are expected to be able to lead and perform a project in a busy FY1 year when we are trying to cope with everything else [Respondent 10 - C]

I am not sure that teaching on serious incident reporting and QI [quality improvement] skills would have necessarily helped in day to day practice; I think this is something that can be learned on the job and from senior colleagues. [Respondent 23 - LC]

#### Curriculum items rated similarly across all confidence groups

##### Important but well met needs: history and examination skills

Interestingly, many items within Communication Skills and Clinical Competencies themes, particularly ‘History Taking’ (n = 0) and ‘Assessing Acutely Unwell Patients’ (n = 1) were ranked infrequently across all groups. Many participants expressed that these items are important but already well met within current undergraduate medical curricula.

We had plenty of history taking and handover training which was helpful. We also had the knowledge to recognise the “acute presentations” when patients were really unwell. These were the easy ones, you knew you needed senior help and you got it pretty fast. The training that was missing was what to do when you aren’t sure whether the patient is “really sick” or not [Respondent 12 - MC]

##### Communicating with other professionals: an unmet need

Items under the theme Communication Skills were rarely identified as unmet in any confidence groups. The only communication skill frequently rated as an unmet need across all groups was that with ‘Difficult Colleagues’ (n = 11, MR 4).

I was lucky that my undergraduate course had a lot of role plays in the final year on difficult relatives, patients, colleagues … It was only after graduating that I realised how important all this was … Valuable learning but I probably didn’t appreciate it at the time as I was so exam focused [Respondent 21 - LC]

I have highlighted the difficult colleagues as most important as I felt this had the greatest impact on my work during FY1 and still find it difficult to this day [Respondent 8 - C]

##### The realities of working life: more preparation needed

Among the most frequently ranked items were those within the Managing Working Life theme. ‘Work-life Balance’ (n = 12, MR 6), ‘Wellbeing’ (n = 14, MR 5.5) and ‘Out of Hours Lifestyle’ (n = 16, MR 7) were all ranked by greater than half of participants as an unmet need. ‘Out of Hours Lifestyle’ was the most frequently ranked unmet need across all themes. Furthermore, 7 participants (across all confidence groups) ranked items in the Managing Working Life theme as their most important unmet need.

Well-being was something that was never ever spoken about. It seems crazy that we were dropped in to intensive on call rotas with little support at times and no one had ever said ‘this could impact on your mental well-being [Respondent 10 - C]

Mental health and coping with working shift patterns/work-life balance were not discussed when I was at medical school - Q&A sessions would have been invaluable prior to starting FY1 [Respondent 23 - LC]

##### Ward-craft

Other items ranked frequently by all groups were those assigned to Job Practicalities, including ‘Referrals’ (n = 16, MR 3) and ‘IT Systems’ (n = 12, MR 4). Participants felt that these skills were crucial to their preparedness for FY1 (high median rank) and are often not taught within undergraduate curricula.

The most helpful thing would be understanding the logistical aspects of working in a hospital. Who to call, what info to prepare etc. This is what I struggled with most. [Respondent 13 - C]

Learning trust processes added efficiency to knowledge; without efficiency, it felt like competency was continually questioned by simple tasks like prescribing or discharge paperwork [Respondent 3 - HC]

I felt most unprepared for the hands-on stuff, despite having lots of “hands on time” at university. It was the stuff I always saw but never actually got to do - making referrals, answering bleeps, working on-call. Being involved in this with a degree of responsibility would have been a big help. FY1 is really difficult at times. [Respondent 10 - C]

‘Referrals’ was the item most frequently unmet across all confidence groups and generally perceived to be of high importance for preparedness (n = 15, MR 3).

I just think that referrals to seniors is done poorly, with a bit of training can be improved very quickly – putting patient safety at the forefront. [Respondent 4- HC]

#### Curriculum agendas

**Figure 3.**
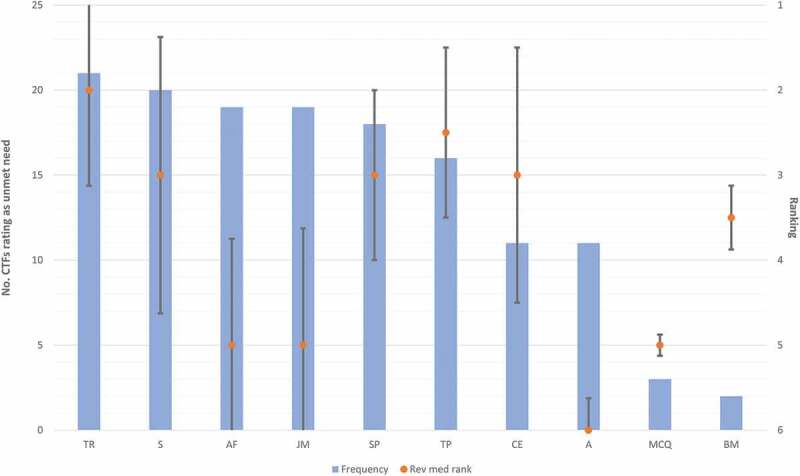
Curriculum agendas

Of the ten curriculum agendas presented to participants, the median number rated as ‘agreed’ was 6 (inter-quartile range 5–7). Most participants seemed aligned on their views, with ‘agreed’ agendas generally sorted with high frequency and uniformly (narrow inter-quartile range). (Table 3 and Figure 3) illustrate the frequency, median rank and interquartile range for each curriculum agenda.

##### Confidence groups’ views of curriculum agendas

There were no significant differences across confidence groups in the median number of curricular agendas selected (median number was 5.5–6.5 across all confidence groups). Agendas were ranked similarly across confidence groups other than ‘Longer Transition Periods’ which was ranked more frequently and with higher importance in the low confident group (n = 5/5, MR 1) compared to the very confident group (n = 2/6, MR 6).

I feel in my last year, shadowing an FY1 for 3 months taught me more about how to cope in the job than I had learned across medical school. [Respondent 24 – LC]

##### Simulation and shadowing: valued agendas

‘Simulation Based Learning’ (n = 20, MR 3) and ‘More Time Working in FY1 Shift Patterns’ (n = 18, MR 3) were frequently and highly ranked across all participants. The strength of simulation-based learning in addressing interpersonal and other human factors was valued in allowing participants to ‘*explore many of the gaps (such as conflict with seniors, patients or uncertainty) in a realistic but controlled environment*’ *[Respondent 14 – MC]*. They also felt that working shift patterns alongside FY1 doctors allowed them to learn the practicalities of the job which may not have been taught elsewhere, however they expressed caution in adding teaching to the responsibilities of already busy junior doctors.

More time working with FY1s … needs to be considered in terms of added responsibility on a likely already overwhelmed FY1 [Respondent 17 - MC]

The highest and most frequently ranked agenda was ‘Transition of Responsibility with Graded Responsibilities’ (n = 21, MR 2). Transitioning responsibility while shadowing was felt to profoundly impact preparedness.

I was lucky to have a transition period, but did not have any responsibility. The shift from zero to full responsibility as you start work is scary [Respondent 10 - C].

The frequency of agreement with a curriculum agenda, and the ranked importance of that agenda were not always aligned. For example, ‘Assessment Aligned with Foundation Competencies’ (n = 19, MR 5) and ‘Junior Doctor Mentors’ (n = 19, MR 5) were frequently agreed to be useful to improve preparedness but typically ranked as less important.

##### Traditional curriculum agendas: low impact

Traditional curriculum items and assessment structures were considered the least important to improve preparedness with ‘Biomedical Teaching’ (n = 2) and ‘Knowledge Based Multiple Choice Questions’ (n = 3) ranked with lowest frequency. Some participants felt that these items took them away from clinical environments and hence negatively impacted preparedness.

I actually think all the multiple choice questions on weird bits of biomedical knowledge stopped me getting to grips with learning to be a good doctor. You can’t learn those answers from being on the wards - you have to go to the library. [Respondent 5 - VC]

## Discussion

### Summary of findings

Our focus groups identified 28 discrete ‘curriculum items’ and 10 ‘curriculum agendas’ that CTFs felt needed to change to better support medical graduates in transitioning to clinical practice. The most frequently rated unmet needs, as rated through a follow-up survey, related to adjusting to managing working life (e.g., wellbeing, learning to work nights/shifts) and becoming familiar with job practicalities (e.g., making referrals, using IT systems). These were described as not covered or even discussed within participants’ undergraduate training. The most highly ranked unmet needs in terms of importance related to clinical decision making and task prioritisation, where participants described a lack of responsibility as an undergraduate impacting on their ability to acquire these skills. Participants felt that being a competent FY1 doctor was as much about efficiency as knowledge, and that it was unrealistic to expect competency from day one. Higher confidence participants described other unmet needs, relating to professional competencies such as quality improvement, teaching and responding to adverse incidents, however these were consistently ranked as low importance. Lower confidence participants did not identify these professional competencies as unmet needs, describing clinical competencies as a more pressing priority. Eliciting a history, assessing/examining patients and prescribing were described as important but well met needs, however clinical decision making and communicating with difficult colleagues were important unmet needs. Having a graded transition of responsibility was the most frequently rated and highest ranked curricular agenda. There was agreement that the assessment culture was too heavily focused on biomedical knowledge rather than the competencies of a working doctor.

### Implications and applications in relation to the existing literature

Transition points have vital implications for curriculum design which is reflected by the large body of literature exploring this topic. Much of the literature on transitions in higher education views them as problems to be mitigated through preparation, hence the term ‘preparedness for practice’. Commentary by O’Brien (2018) suggests we reframe our perspective: rather than viewing transitions as problems to solve or remove, we should instead consider them as opportunities to learn new behaviours, confidence, and skills. Further, Colbert-Getz et al. (2016) proposes a theoretical approach grounded on the unavoidable nature of transition, and the requirement for targeted interventions that respect individuals’ experience to optimise learning and minimise emotional stressors. In our study, even participants who felt highly prepared at the time of graduation reported a 1 to 2-month delay in having full confidence in conducting their clinical role, confirming the inevitability of challenges despite best preparation. Colbertz-Getz et al. (2016) use cognitive load theory to explore three key contributors to transitional learning: personal characteristics, task readiness and contextual factors. Our research identifies unmet curricular items and agendas that map across these three domains with clear impacts on transitional experiences. Whilst it may not be possible to address personal or contextual factors outside the academic institutional remit, we believe that medical schools could do more to support this important transition. Our findings provide valuable insights into what these changes might be, with a clear requirement that interventions systematically address important unmet needs including personal wellbeing, the communicational and relational aspects of work, situational and contextual ‘ward-craft’, and effective clinical decision making.

Kilminster et al. ([18) suggest that transitions should be viewed as ‘critically intensive learning periods’. This was reflected in our findings where participants saw the transition as a learning curve and rated ‘graded transition towards clinical responsibility’ as their most important curriculum agenda. Graded transition is not a new concept, and much work has been done to describe how entrustment can be based on competency and how entrustment decisions may be effectively facilitated [[19]. Internationally, medical schools increasingly offer extended periods of shadowing following final examinations, known as assistantships, to support students in transitioning into the workplace [20,21]. There is however evidence to suggest that assistantships are not always helping students to develop the clinical competencies that they need, and rather preoccupy students with routine tasks such as discharge summaries or chasing results [22]. Our research suggests that more active participation in clinical decision making is needed, and that curricular reform should expose students to appropriately graded and supported clinical responsibility earlier in their undergraduate learning. Previous research also suggests that clinical responsibility may lessen the social burden of trying to ‘fit in’ with clinical teams, while enhancing the development of professional attitudes and skills [1].

Many of the items identified by participants as unmet needs related to complex communicational and relational aspects of work, for example negotiating a referral with a busy colleague, or explaining end-of-life care to an upset relative. Assessment design reliant on multiple choice questioning or objective structured tests of clinical competence is unlikely to give this complex, uncodifiable knowledge the curricular emphasis it needs. We suggest that this may be one reason for their de-prioritisation in undergraduate curricula, despite their salience to preparedness. Interestingly, participants felt that the prevailing assessment paradigm was driving students away from learning in the clinical workplace and may therefore be having a negative impact on preparedness. This supports a move towards more complex low-stakes assessment instruments which may not individually be reliable, but when taken together give an accurate picture of both engagement and attainment [23]. This is particularly interesting in the context of the proliferation of medical licensing assessments internationally [[24]. These aspire to address variation in undergraduate assessment design and provide reassurance of standardised minimum competency [25]. However, they are unlikely to address key areas of preparedness [5,7,26] and may undervalue or even undermine the acquisition of what Eraut calls ‘tacit knowledge’ that occurs through immersion in the workplace [27]. Our research suggests that medical schools could do more to support the acquisition of more complex clinical competencies through lower-stakes assessments and assignments that focus on authenticity and growth.

Many of the curricular items identified as unmet needs relate to what Fenwick calls ‘sociomaterial knowledge’ that is situated in its context, for example local protocols, social conventions and systems [28]. We suggest that medical schools could do more to support students in developing strategies to acquire this type of knowledge.

Our research suggests that higher professional competencies (career planning, quality improvement, responding to adverse incidents, teaching) are only valued by learners that are already confident in their clinical competencies. More research is needed to establish whether it is better to teach these higher professional competencies earlier in the curriculum when learners are less pressured, or after core clinical competencies have been achieved. A graded transition might also provide protected time for the development of higher professional competencies during student assistantships.

### Strengths and weaknesses

A strength of this research is that it is based on the experiences and insights of CTFs who are both experts through experience (as recent graduates), and experts through training (as educators). The sample size was small so statistical inference should be taken with caution. However, we have described notable patterns in the data relating to the types of competencies that were highly ranked unmet needs, and which were differentially important to confident and under-confident graduates. Heterogeneity between participants, including variance in year of graduation and course type, may confound differences in perceptions and may not necessarily reflect more recent curricular advances. As this study is situated in a specific national context, the results will need to be interpreted with an appreciation for locally relevant contextual factors.

## Conclusions

Our research suggests that successful transition between medical school and newly qualified doctor could be enhanced by 1) graded entrustment; 2) enhancing shadowing opportunities and assistantships through strategies for active learning; 3) recognising the importance of complex clinical competencies (beyond history and examination) and developing appropriate strategies for teaching and assessment that focus on authenticity and growth; 4) recognising that wellbeing and learning to manage working life are part of preparedness for practice 5) articulating the importance of local sociomaterial ‘ward-craft’ and giving students the tools to acquire it; and 6) considering how to position and balance higher professional competencies in relation to clinical competencies.

## Data Availability

The datasets used and/or analysed during the current study are available from the corresponding author on reasonable request.

## Appendix 1

Qualtrics Survey: https://imperialcollegelondon.box.com/s/sldd46gkqxsh9nv60zut0zddu5p8qome
